# Whole-genome sequencing reveals selection signatures associated with important traits in six goat breeds

**DOI:** 10.1038/s41598-018-28719-w

**Published:** 2018-07-10

**Authors:** Jiazhong Guo, Haixi Tao, Pengfei Li, Li Li, Tao Zhong, Linjie Wang, Jinying Ma, Xiaoying Chen, Tianzeng Song, Hongping Zhang

**Affiliations:** 1 College of Animal Science and Technology, Sichuan Agricultural University, Chengdu, Postcode 611130 China; 2 Institute of Animal Science, Tibet Academy of Agricultural and Animal Husbandry Science, Lhasa, 850009 China

## Abstract

Comparative population genomics analysis is an effective approach to identify selection signatures in farm animals. In this study, we systematically investigated the selection signatures in six phenotypically diverse goat breeds using SNPs obtained from pooled whole-genome resequencing data. More than 95.5% of 446–642 million clean reads were mapped to the latest reference goat genome, which generated a sequencing depth ranging from 22.30 to 31.75-fold for each breed. A total of 5,802,307, 6,794,020, 7,562,312, 5,325,119, 8,764,136, and 9,488,057 putative SNPs were detected in Boer, Meigu, Jintang Black, Nanjiang Yellow, Tibetan, and Tibetan cashmere goats, respectively. Based on the genome-wide F_ST_ and expected heterozygosity scores along 100-kb sliding windows, 68, 89, 44, 44, 19, and 35 outlier windows were deemed as the selection signatures in the six goat breeds. After genome annotation, several genes within the selection signals were found to be possibly associated with important traits in goats, such as coat color (*IRF4*, *EXOC2*, *RALY*, *EIF2S2*, and *KITLG*), high-altitude adaptation (*EPAS1*), growth (*LDB2*), and reproduction traits (*KHDRBS2*). In summary, we provide an improved understanding of the genetic diversity and the genomic footprints under positive selection or the adaptations to the local environments in the domestic goat genome.

## Introduction

As an important livestock species distributed throughout the world, goats provide abundant meat, milk and fiber for human consumption, particularly in the developing countries of Asia and Africa^1^. To date, domestic goats have evolved into many distinct breeds (e.g., dairy, meat and fiber breeds), mainly as a result of long-term artificial selection^2^. In turn, this selection might have left genetic footprints in the goat genome, reflecting a phenotypic evolution driven by different breeding objectives or the adaptation to the local environments.

Genome scans based on population genetics statistics (e.g., F_ST_) are an effective approach to detecting genes under positive selection for populations without known phenotypes^3–5^. During the past several years, many studies on the genome-wide identification of selection signals have emerged in pigs^6^, cattle^7–9^, sheep^10–12^, and goats^11,13^, based on SNP chips. These results have demonstrated how positive selection acting on complex and Mendelian traits has changed the genetic composition of domestic animals. However, ascertainment bias in SNP genotype calling can lead to skewed allele frequency distributions, thus affecting the accuracy of population genetic analyses^14^. For example, the SNPs on the Goat SNP50 BeadChip were discovered in six Saanen, seven Alpine and three Creole goats^15^. In addition, the density of this chip was too limited to obtain more precise selection mapping.

Whole-genome sequencing approaches with pooled DNA have recently developed into a powerful tool to systematically identify selection signals at a relatively low cost^16^, particularly for non-model organisms. In livestock, a pioneering study that used the pooled genome sequencing^17^ reported a number of putative selective sweeps under intensive artificial selection in broiler and layer chickens, suggesting rapid phenotypic evolution in domestic animals. Similarly, Rubin *et al*. revealed many selection signals and structural variations in 24 pig populations from across the world^18^, which further demonstrated the utility of pooled genome resequencing. Whole-genome sequencing substantially facilitates comparative genomics studies in goats since there is no high-density goat SNP chip^15,19^. Based on genotype-by-sequencing, Wang *et al*.^20^ reported several genes under positive selection in eight goat populations, such as *FGF5* for cashmere traits, *ASIP* for coat color, and *NOXA1* for high-altitude adaptation. The genomic comparisons of Dazu black and Inner Mongolia cashmere goats showed that the selective sweep regions were related to reproduction and production traits^21^. Moreover, exome sequencing of 330 cashmere goats uncovered genetic differentiation induced by high-altitude adaptations in the Tibetan cashmere goat^22^, which supported the function of *EPAS1* in response to hypoxia and strong selection pressure for its locus. However, the selection signals associated with economically important traits in goats remain largely unknown, including coat color, milk production and reproduction traits.

Southwest China has a number of domestic goat populations that are raised in different ecoregions (differing in temperature, humidity, and altitude) and have been subjected to different selection goals. For example, the Nanjiang Yellow goat is a breed that was recently developed for meat production in Southwest China. The coat color of this breed is yellow and black, with a notable black stripe on the back. In terms of reproduction performance, the indigenous Meigu goat in Sichuan, with its black coat, is a prolific breed (average 2.07 lambs per parity) with precocious puberty (the onset of puberty at 2–3 months of age). Additionally, the Jintang Black goat is an indigenous Chinese goat breed with a solid black coat. Compared to low-altitude breeds, the Tibetan goat is deemed as an indigenous Chinese goat breed that lives on the Qinghai-Tibetan plateau but also includes populations from different ecoregions. For instance, the Tibetan cashmere breed from Cuoqin County lives at very high altitudes (average ~4700 meters) and generally shows a black coat. Moreover, the Boer, which originated in South Africa, is the most renowned meat goat breed, with a large body size and a fast growth rate. In this study, we applied pooled whole-genome sequencing to reveal genetic loci under artificial and natural selection in Boer, Meigu, Jintang Black, Nanjiang Yellow, Tibetan, and Tibetan cashmere goats.

## Materials and Methods

### Ethics statement

In this study, all experiments involving animals were performed in accordance with the guidelines and regulations for the Administration of Affairs Concerning Experimental Animals (Ministry of Science and Technology, China). All experimental protocols were approved by the Institutional Animal Care and Use Committee of the College of Animal Science and Technology, Sichuan Agricultural University, Sichuan, China (No. DKYB20081003).

### Animals and whole-genome sequencing

In this study, a total of six domestic goat breeds were included: The Boer goat (BE) from Jianyang, the Nanjiang Yellow goat (NJ) from the Nanjiang Yellow goat breeding farm in Nanjiang County, the Meigu goat (MG) from Meigu County, the Jintang Black goat (JT) from the Jintang Black breeding farm in Jintang County, the Tibetan goat (TG) from the Liangshan Prefecture in Sichuan, and the Tibetan cashmere goat (TC) from Cuoqin County in Tibet. For high-throughput sequencing, we extracted genomic DNA samples from the whole blood samples of 20 animals per breed. DNA was mixed into a single pool for each breed using the same amount per individual. Six DNA libraries with insert sizes of approximately 350 bp were constructed following the manufacturer’s instructions, and 150 bp paired-end reads were generated using the Illumina HiSeq X10 platform.

### Alignment of reads and SNP calling

After trimming the adapters, raw reads were filtered for base quality using Trimmomatic^23^ (v0.36) with the following parameters: LEADING:20, TRAILING:20, SLIDINGWINDOW:4:20, and MINLEN:50. The filtered reads were mapped using the ‘mem’ algorithm of BWA^24^ (v0.7.12) against the goat reference genome^25^ (assembly ARS1). It should be noted, however, that the current goat reference genome does not include the information from Chromosome X. Picard software (v2.10.6) (http://broadinstitute.github.io/picard/) was then applied to remove the duplicated reads, followed by local realignment around existing indels and base quality score recalibration using GATK^26^ (v3.7-0).

Since pooled DNA samples were sequenced in this study, we performed SNP calling using VarScan^27^ (v2.4.3) with the following parameters: min-coverage 15, min-reads2 2, and min-avg-qual 30. The SNPs were finally retained based on a *P*-value < 0.05 (the significance of the read counts supporting each allele *vs*. the expected distribution based on sequencing error alone) and minor allele frequencies (MAF) ≥ 0.025, considering that DNA samples of 20 individuals were pooled in each of the sequence libraries.

### Principal component analysis and detection of selection signals

GCTA^28^ software (v1.26.0) was applied to conduct principal component analysis (PCA) after data transformation of SNPs in PLINK PED format via PLINK^29^ (v1.07) to examine the genetic relationships among the six goat breeds.

In this study, two approaches (F_ST_ and expected heterozygosity) were utilized to identify the selection signatures in the genomes of domestic goats. First, the weighted population pairwise F_ST_ values were calculated for each SNP based on ${F}_{ST}=\frac{{s}^{2}}{\bar{p}(1-\bar{p})+\frac{{s}^{2}}{r}}$, where *s*^2^ represents the sampling variance of allele frequencies between two populations, $\bar{p}$ represents the overall average allele frequency across populations, and *r* = 2 represents the number of populations^30^. The F_ST_ values were then averaged over SNPs using a 100-kb sliding window (≥30 SNPs) with a 25-kb step size for each comparison. For a given breed, the final F_ST_ value was the overall mean of each window with the same genomic coordinates across five comparisons. In addition, the expected heterozygosity (*H*_*p*_) was used to scan selection signals. Specifically, *H*_*p*_ values of individual SNPs were first calculated according to ${H}_{P}=\,2{\sum }^{}{n}_{maj}{\sum }^{}{n}_{min}$, where ${\sum }^{}{n}_{maj}$ and ${\sum }^{}{n}_{min}$ represent the sums of the numbers of the major and minor alleles at each locus, respectively. The *H*_*p*_ values were further averaged along 100-kb sliding windows with a step size of 25 kb. To detect selection signals, the F_ST_ and H_p_ values were then Z-transformed as follows: ${{\rm{ZF}}}_{\mathrm{ST}}=\,\frac{{{\rm{F}}}_{{\rm{ST}}}-{{\rm{\mu }}F}_{{\rm{ST}}}}{{{\rm{\sigma }}F}_{{\rm{ST}}}}$ and ${{\rm{ZH}}}_{{\rm{P}}}=\,\frac{{{\rm{H}}}_{P}-{{\rm{\mu }}H}_{P}}{{{\rm{\sigma }}H}_{{\rm{p}}}}$. Finally, the top 0.5% of windows showing both extremely high F_ST_ values (the top 0.5% of ZF_ST_ distributions) and extremely low H_p_ scores (the bottom 0.5% of ZH_p_ distributions) were proposed to be under selection (i.e., candidate selection regions).

Based on genome annotation, a gene was deemed to show evidence of being under selection if it overlapped with an outlier genomic window based on both ZF_ST_ and ZH_p_ values. To better understand the molecular functions of the genes that overlapped with the candidate signatures of selection, we performed functional enrichment analysis using PANTHER^31^ (http://www.pantherdb.org/). Because the goat genome information is currently unavailable in PANTHER, human homologous gene symbols were used.

### Accession codes

All raw high-throughput sequence data in the current study are available from the NCBI SRA database (accession number(s) PRJNA407657).

## Results and Discussion

### Abundant genetic variation detected in the six goat breeds

**Figure 1:**
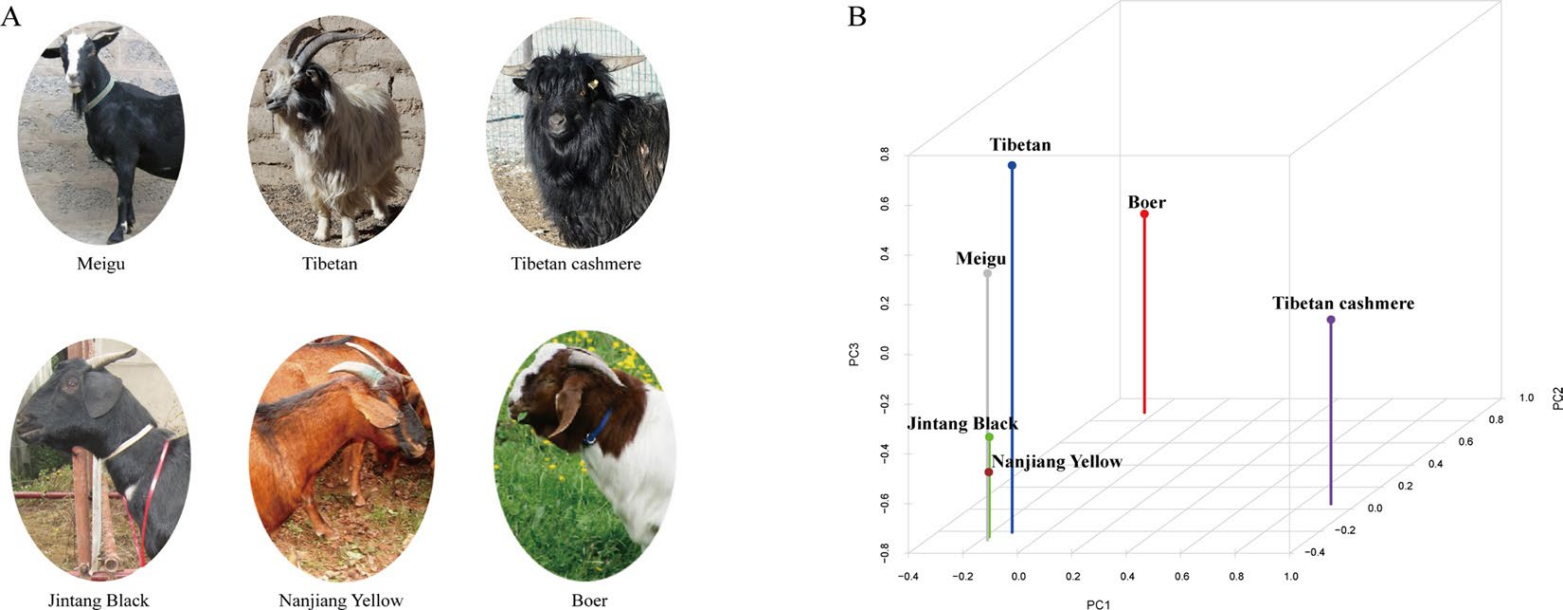
Summary of all six domestic goat breeds included in this study. (**A**) Pools of genomic DNA from the following populations were sequenced: Boer (n = 20; sex = F/M), Meigu (n = 20; sex = F), Jintang Black (n = 20; sex = F/M), Nanjiang Yellow (n = 20; sex = F), Tibetan (n = 20; sex = F/M), and Tibetan cashmere goat (n = 20; sex = M). Photographs were taken by Hongping Zhang and Tianzeng Song. (**B**) Plot of the first three principal components (PC1, PC2, and PC3) of six goat breeds based on the detected SNPs.

The main purpose of this study was to systematically identify the selection signals underlying phenotypic evolution in domestic goats by exploiting the SNPs obtained via pooled whole-genome DNA resequencing data (Fig. 1A). Here, genome sequencing yielded a total of 478 Gb of paired-end raw reads with a length of 150-bp from six goat breeds, and approximately 446–642 million clean reads were obtained per breed after quality control (Supplementary Table S1). More than 95.50% of the total clean reads were mapped against the latest goat reference genome (assembly ARS1) with a coverage of ~99.88%. These mapped reads also generated an average sequencing depth of 26.0 × per breed, ranging from 22.30 (NJ) to 31.75 (TG) fold (Supplementary Table S1), indicating that high quality sequences were obtained in this study.

**Figure 2:**
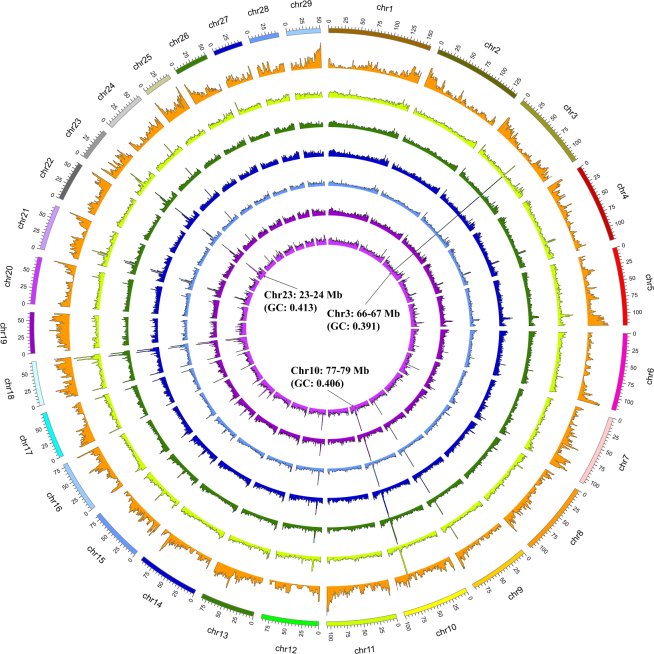
Genome-wide histograms of SNPs detected in six goat breeds. The outermost circle represented GC content of the goat reference genome sequence. The circles from outer to inner show the genome-wide distribution of SNPs (counted in 1-Mb non-overlapping windows) in Boer, Meigu, Jintang Black, Nanjiang Yellow, Tibetan, and Tibetan cashmere goats, respectively.

Based on these stringent thresholds, a total of 15,215,715 unique SNPs were identified among the six goat breeds. As expected, the largest number (9,488,057) of SNPs was detected in Tibetan cashmere goat (Table 1) followed by the Tibetan goat (8,764,136), which likely reflects a larger genetic difference between both highland breeds and the breed used for the reference genome (San Clemente). In contrast, the Nanjiang Yellow goat displayed the lowest number of SNPs (5,325,119). These high quality SNPs resulted in a high density of 2.16–3.85 SNPs/kb and similar genome-wide distributions among all six goat breeds (Table 1). Such a density of SNPs enabled an accurate search for selection signals under artificial and natural selection in the six goat breeds. Strikingly, high polymorphic regions (e.g., an average density of >15 SNPs/kb) were present on several chromosomes, such as 66–67 Mb on chromosome 3, 77–79 Mb on chromosome 10, and 23–24 Mb on chromosome 23 (Fig. 2). However, 107 of the 125 annotated genes in these three genomic regions were identified by LOC symbols; thus, they were not associated with known genes, which hindered further investigation into the biological implications. Additionally, the GC content was not low (0.391, 0.406, and 0.413) for the three genomic regions (Fig. 2) compared to the overall mean value (0.427) for the reference genome (assembly ARS1)^25^. Table 1 shows that the average MAF across all SNPs was similar (0.297–0.313) among all six breeds. In addition, the overall average heterozygosity of all SNPs ranged from 0.388 (TG) to 0.403 (TC) among the six breeds, indicating abundant genetic diversity.

**Table 1:** Summary of genome-wide SNPs in six goat breeds.

Breed	SNPs			
	Number	SNP/1 kb	MAF	H_p_
Boer	5,802,307	2.35	0.312	0.399
Meigu	6,794,020	2.75	0.311	0.399
Jintang Black	7,562,312	3.07	0.307	0.397
Nanjiang Yellow	5,325,119	2.16	0.312	0.399
Tibetan	8,764,136	3.55	0.297	0.388
Tibetan cashmere	9,488,057	3.85	0.313	0.403

The results of PCA clearly classified our six goat breeds based on the identified SNPs (Fig. 1B), which implied high genetic differentiation among these breeds. Moreover, the Boer and Tibetan cashmere goats were genetically distinct among these breeds. As expected, relatively close relationships were observed between the Nanjiang Yellow and Jintang Black goats, which is consistent with their neighbouring habitats. Interestingly, a long genetic distance was found between the two Tibetan goat populations, reflecting their different genetic composition. To date, no definition of breed standard exists for domestic goats living on the Qinghai-Tibetan plateau and neighbouring regions. Our previous study showed that Tibetan goats had four genetic origins based on the mitochondrial D-loop sequences of 10 populations^32^. Additionally, such differentiation could be caused by long-term adaptations to the local environments with different oxygen contents, temperatures, and ultraviolet (UV) exposure on the Qinghai-Tibetan plateau.

### Numerous highly differentiated genomic regions in the six goat breeds

**Figure 3:**
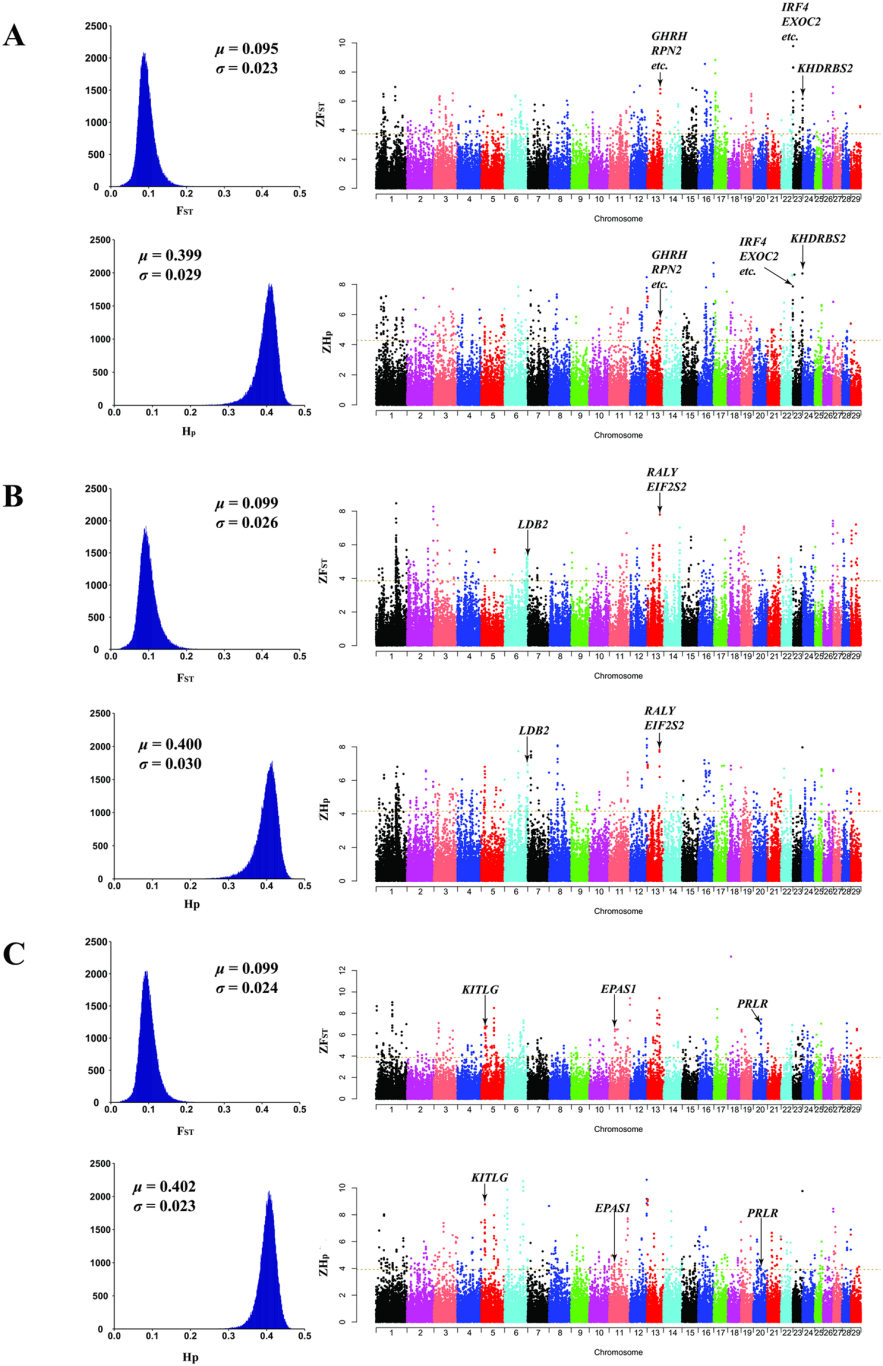
Genome-wide distributions of selection signals in the Meigu (**A**), Nanjiang Yellow (**B**), and Tibetan cashmere (**C**) goat. Manhattan plots of ZF_ST_ (>0) and absolute values of ZH_p_ (<0) across all autosomes were plotted with different colors. The ZF_ST_ and ZH_p_ values were calculated for each sliding 100-kb window with steps of 25 kb across all autosomes. The horizontal dashed line indicates the 99.5 percentile of all the ZF_ST_ or ZH_p_ values.

Although genome scans based on population differentiation (e.g., F_ST_) may give false positive findings, this method is an effective approach to detect selection footprints for populations without known phenotypes^3–5^. According to the results of the PCA, we first calculated F_ST_ values with a sliding 100-kb window with a step size of 25 kb to identify selection signatures in the six goat populations. As expected, Boer goats, which originated from the South Africa, showed the highest overall average F_ST_ value (0.123) (Fig. 3A), which is in accordance with its distant genetic relationship with the five breeds of Chinese origin. The average F_ST_ values were 0.095, 0.093, 0.099, 0.091, and 0.099 for the Meigu, Jintang Black, Nanjiang Yellow, Tibetan, and Tibetan cashmere goats, respectively (Fig. 3B,C, Supplementary Fig. S1), suggesting moderate genetic differentiation among these breeds.

**Figure 4:**
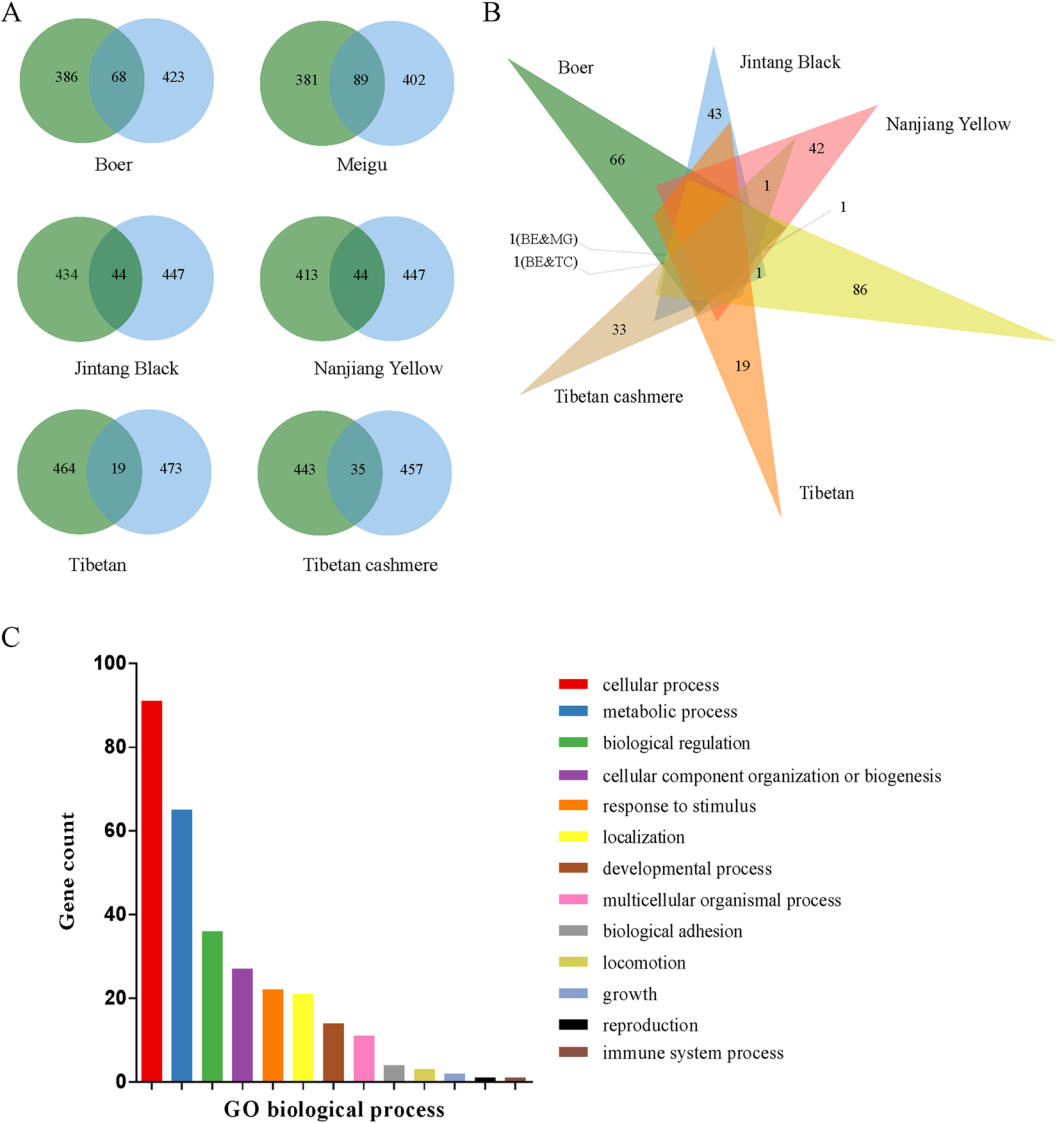
Summary of the overlapped outlier windows detected via ZF_ST_ or ZH_p_ scores. (**A**) A Venn diagram of the outlier windows detected via ZF_ST_ or ZH_p_ scores in six goat breeds, based on their genomic positions. Green circles represent the numbers of the outlier windows detected via ZF_ST_ values, while the light blue circles represent the windows identified via ZH_p_ scores. (**B**) A Venn diagram of the overlapped outlier windows across all the six goat breeds, based on their genomic positions. (**C**) Summary of the biological processes into which the candidate genes can be classified.

Only the top 0.5% of windows with high average ZF_ST_ values were determined as selective sweep regions in the empirical distributions for each breed to reduce false positives. Accordingly, 454, 470, 478, 457, 483, and 478 outlier windows (ZF_ST_ ≥ 3.72, 3.75, 3.68, 3.86, 3.44, and 3.88; corresponding F_ST_ ≥ 0.26, 0.18, 0.17, 0.20, 0.16, and 0.19) were detected in the Boer, Meigu, Jintang Black, Nanjiang Yellow, Tibetan, and Tibetan cashmere goats (Fig. 4, Supplementary Dataset S1), respectively. The windows with the highest F_ST_ values within individual breeds were located at 1.95–2.05 Mb on chromosome 4 (ZF_ST_ = 7.282, F_ST_ = 0.397), 0.20–0.30 Mb on chromosome 23 (ZF_ST_ = 9.782, F_ST_ = 0.320), 37.50–37.60 Mb on chromosome 6 (ZF_ST_ = 9.668, F_ST_ = 0.301), 102.73–102.83 Mb on chromosome 1 (ZF_ST_ = 8.466, F_ST_ = 0.319), 79.85–79.95 Mb on chromosome 7 (ZF_ST_ = 8.571, F_ST_ = 0.259), and 16.075–16.175 Mb on chromosome 18 (ZF_ST_ = 13.307, F_ST_ = 0.418) for Boer, Meigu, Jintang Black, Nanjiang Yellow, Tibetan, and Tibetan cashmere goats (Fig. 3, Supplementary Fig. S1 and Dataset S1), respectively.

### Many highly differentiated windows also show relatively low heterozygosity values

We calculated the Z-transformed heterozygosity (ZH_p_) with 100-kb sliding windows (with 25 kb steps) to scan the selection signatures in the domestic goat genomes. The overall average H_p_ values across all the windows were 0.399, 0.399, 0.397, 0.400, 0.388, and 0.402 in the Boer, Meigu, Jintang Black, Nanjiang Yellow, Tibetan, and Tibetan cashmere goats, respectively (Fig. 3, Supplementary Fig. S1). Similarly, only the bottom 0.5% of windows with the lowest ZH_p_ scores (ZH_p_ ≤ −4.107, −4.285, −4.208, −4.163, −3.919, and −3.918) were deemed as selection regions, which led to ~491 outlier windows per breed (Fig. 4A and Supplementary Dataset S2).

Only the overlapping windows that were detected via both approaches were further considered as putative selection signatures in each breed to improve the confidence for the identified selection signatures. Accordingly, there were 68, 89, 44, 44, 19 and 35 outlier windows in Boer, Meigu, Jintang Black, Nanjiang Yellow, Tibetan, and Tibetan cashmere goats, respectively (Fig. 4A). Comparing their genomic locations showed that most of these selection signals were breed-specific (Fig. 4B), reflecting distinct phenotypic evolutions under different selection objectives or adaptations to the local environments.

According to reference genome annotation, a total of 49 (Boer), 85 (Meigu), 56 (Jintang Black), 32 (Nanjiang Yellow), 29 (Tibetan), and 18 (Tibetan cashmere) genes were found in the putative selection signals of each breed (Supplementary Tables S2–S7), respectively. However, some candidate selection regions (e.g., chr1: 30.725–31.0 Mb for the Boer) were annotated without any genes, due to the incomplete genome annotation (Supplementary Dataset S3 and Tables S2–S7). Although none of the total candidate genes were significantly enriched in any biological processes, these genes could be classified into 13 biological processes, such as cellular process (GO:0009987), metabolic process (GO:0008152) and biological regulation (GO:0065007). Based on their biological functions and information from published studies, several genes were possibly responsible for the important traits in goats and are thus presented in greater detail below.

### Three known genetic loci are possibly associated with coat color in goats

**Figure 5:**
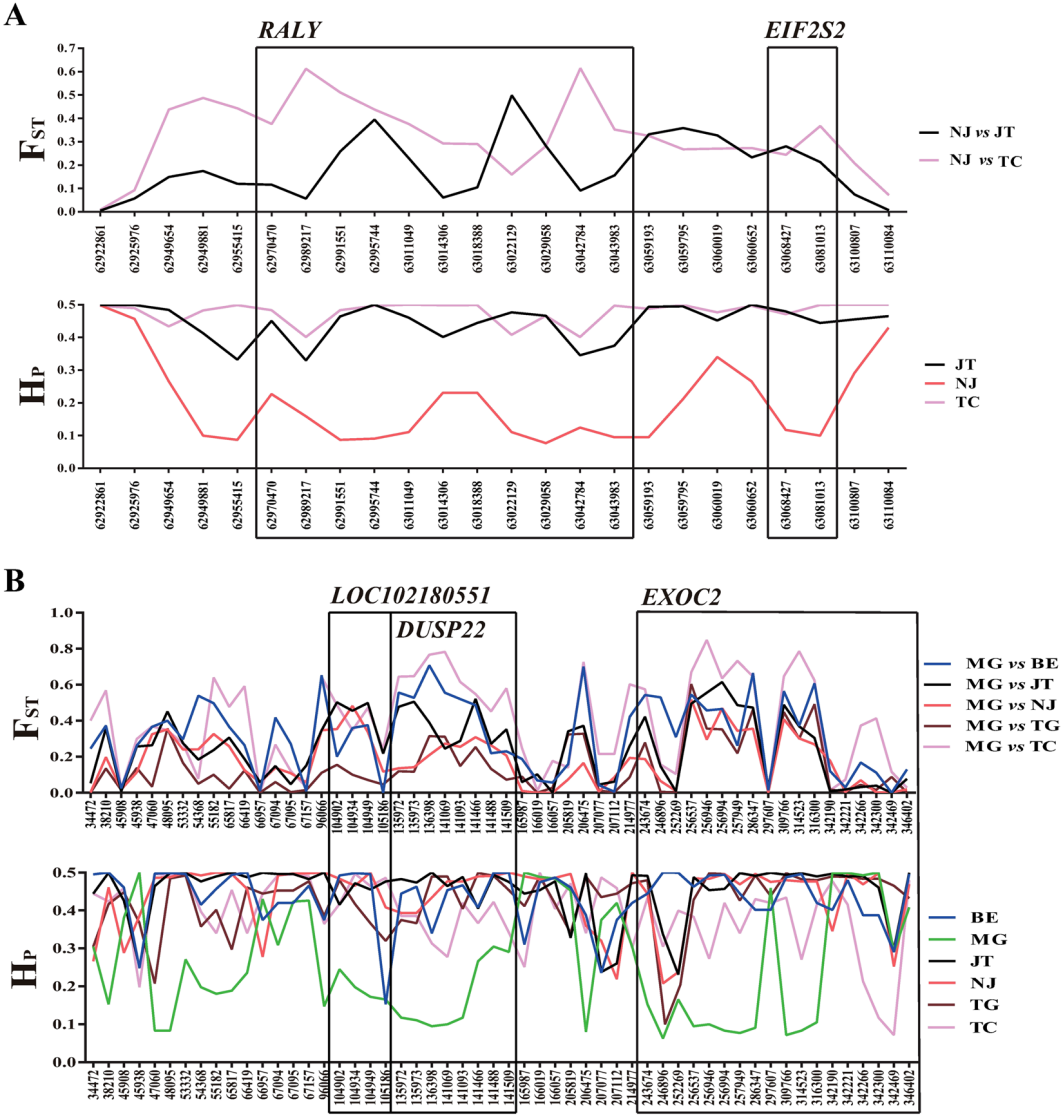
Summary of F_ST_ and ZH_p_ values of each SNP at the *RALY-EIF2S2* and *IRF4-EXOC2* loci. (**A**) The *RALY-EIF2S2* locus; (**B**) the *IRF4-EXOC2* locus.

Coat color is one of the most important phenotypic features in contemporary livestock breeds. As shown in Fig. 1A, coat color varied among all six goat breeds included in this study. Accordingly, we identified three known genetic loci that were previously associated with skin or hair color variations in humans^33,34^. Specifically, a highly differentiated region (ZF_ST_ = 4.100, F_ST_ = 0.205) at 63.00–63.10 Mb on chromosome 13 encompassed the genes *RALY* (*RALY heterogeneous nuclear ribonucleoprotein*) and *EIF2S2* (*eukaryotic translation initiation factor 2 subunit 2*) in the Nanjiang Yellow goat (Fig. 3B, Supplementary Table S5 and Dataset S2). Compared to the two black-coated breeds (i.e., the Jintang Black or Tibetan cashmere), four SNPs (chr13: 62,989,217, 62,995,744, 63,022,129, and 63,042,784) in the introns of *RALY* had very low H_p_ values (~0.1) but high F_ST_ scores (>0.3) in the Nanjiang Yellow goat (Fig. 5A). Notably, the genomic location of *RALY* and *EIF2S2* is close to that of *ASIP* in the goat genome, although *ASIP* was not detected as a selection gene in the Nanjiang Yellow goat. *ASIP* encodes an agouti signalling protein that promotes hair follicle melanocytes to synthesize pheomelanin (i.e., a yellow pigment) in animals^35^. The lethal agouti-yellow mutation not only deletes *RALY* and *EIF2S2* but also causes the ectopic expression of *ASIP* in the mouse^36^; the expression levels of *ASIP* and *RALY* could be regulated by a single 5′ UTR region in this species^37^. Furthermore, one SNP close to the *EIF2S2-ASIP* region was found to be associated with skin color in African-admixed humans^38^. In dogs, a 16-bp duplication in *RALY* was significantly associated with the saddle tan and black-and-tan phenotypes in Basset Hounds and Pembroke Welsh Corgis^39^. Strikingly, the Nanjiang Yellow goat generally has a yellow or tan coat color, with a particular black stripe on the back (Fig. 1A). Therefore, our results indicated the *RALY-EIF2S2-ASIP* locus is a putative genetic locus that influences the skin and hair pigmentation in the Nanjiang Yellow goat.

In the Meigu goat, the starting region (0.025–0.35 Mb) of chromosome 23 had the highest F_ST_ values (ZF_ST_ = 9.782, F_ST_ = 0.320) and low Hp scores (ZH_p_ = −6.965, H_p_ = 0.197) (Supplementary Dataset S3). Based on the genome annotation, this region harbors genes *LOC102180551*, *DUSP22* (*dual specificity phosphatase 22*), *IRF4* (*interferon regulatory factor 4*), and *EXOC2* (*exocyst complex component 2*) (Fig. 3B, Supplementary Tables S3). Previous studies reported that the SNPs located at or between *IRF4* and *EXOC2* were associated with skin pigmentation, hair color or skin sensitivity to the sun in humans^40,41^. Further studies demonstrated that a mutation in the fourth intron of *IRF4* could enhance melanin synthesis by up-regulating the expression of tyrosinase in humans, and mice lacking *IRF4* showed lighter coat color^42^. Since it has been implicated in melanocytic biology, the IRF4 protein was proposed as a diagnostic marker for various melanoma subtypes^43^. However, the sequence of the 4^th^ intron of *IRF4* was not conserved based on BLAST (data not shown), and no plausible SNPs were detected in this gene for the Meigu goat. However, six SNPs (chr23: 256,537, 256,946, 256,994, 286,347, 309,766 and 314,523) in the introns of *EXOC2* had a very low heterozygosity of ~0.1 and very high F_ST_ values of >0.3 between Meigu and the five other breeds (Fig. 5B), indicating that this gene was more likely to be under selection. A recent genome-wide association study (GWAS) demonstrated that one SNP (rs12210050) in *EXOC2* was strongly associated with the tanning ability of Europeans^44^. Another SNP (rs9328342) in this gene was significantly related to serum 25(OH)D concentration in humans^45^. In summary, this genomic region might be associated with coat color or the absorption of 25(OH)D in the Meigu goat. However, follow-up studies are necessary to replicate this finding and investigate the relevant molecular mechanisms.

The region between 18.00 and 18.10 Mb on chromosome 5 in the Tibetan cashmere goat showed high differentiation from the five other breeds (ZF_ST_ = 6.592, F_ST_ = 0.257) and a low heterozygosity (ZH_p_ = −5.388). According to genome annotation, this sweep region contained *KITLG* (*KIT Ligand*), which is involved in the differentiation and migration of melanocytes^46^ (Fig. 3C, Supplementary Table S7). This selection signature was recently detected in Taihang Black goats^20^ and a Moroccan black goat population^47^, which agrees with the black hair of the Tibetan cashmere goat. A molecular study has also revealed a SNP in the enhancer region of *KITLG* increased its expression by interacting with LEF1, thus contributing to the blond hair phenotype of northern Europeans^48^. However, it was established that dark skin could reduce UV-induced photolysis of folate and protect skin cells from exposure to UV radiation, which could lead to sunburn and increase the risk of skin cancer in humans^49^. Similarly, the SNPs in *KITLG* showed a significant, genome-wide association with UV-protective eye area pigmentation in Fleckvieh cattle^50^. Thus, we cannot exclude the possibility that the *KITLG* gene was under selection due to high altitude adaptations or the intensive UV radiation on the Tibetan plateau.

### One selective sweep region is likely related to body size traits in the Nanjiang Yellow goat

Growth rate and body weight are the most economically important traits in livestock that are specialized for meat production. In the Nanjiang Yellow goat (a meat type breed in China), the distant region (112.10–112.20 Mb) of chromosome 6 showed high differentiation (ZF_ST_ = 5.26, F_ST_ = 0.24) and low heterozygosity (ZH_p_ = −4.23, H_p_ = 0.27) and is thus identified as a putative selection signal (Fig. 3B, Supplementary Table S5). Interestingly, this region was part of the gene *LDB2* (*LIM domain-binding factor 2*), which has been identified as a key regulator of transendothelial migration of leukocytes and atherosclerosis^51^. GWAS has shown a significant, genome-wide association with body weight during weeks 7–12 and average daily gain for weeks 6–12 in a chicken F2 resource population^52^. Findings in Beijing-You^53^ and Jinhai Yellow chickens^54^ further supported *LDB2* as a candidate gene for influencing the body weight at different ages in chickens.

### *KHDRBS2* is possibly associated with reproduction traits in the Meigu goat

The 48.175–48.650 Mb region on chromosome 23 was highly differentiated (ZF_ST_ = 6.159, F_ST_ = 0.236) between Meigu and the five other breeds (Fig. 3A, Supplementary Dataset S3). Furthermore, one window of this region had a low ZH_p_ score of −7.121 (H_p_ = 0.193) (Supplementary Dataset S3). According to the genome annotation, this region only encompassed *KHDRBS2*, which has been classified as a reproduction process (GO:0000003) (Fig. 4C). A recent GWAS reported that two SNPs near or within *KHDRBS2* were significantly associated with the number of teats in Large White pigs^55^. Additionally, a SNP near *KHDRBS2* was associated with pregnancy status in Brahman beef cattle^56^.

### *EPAS1* is possibly associated with high-altitude adaptation in the Tibetan cashmere goat

Adaptations to low oxygen, high-intensity UVs and cold temperatures in the Tibetan plateau could significantly change the genetic composition of Tibetan goats. In this study, the region on chromosome 11 (28.31–28.46 Mb) encompassing *endothelial PAS domain protein 1* (*EPAS1*) was detected as a selection signal (ZF_ST_ = 6.329; ZH_P_ = −4.328) in the Tibetan cashmere goat (Supplementary Dataset S3). Recent studies have demonstrated that *EPAS1* was an important candidate gene for the adaptation to low-oxygen environments for humans^57,58^ and dogs^58,59^ on the Tibetan plateau. In goats, Song *et al*.^22^ recently showed that SNPs in genes related to the cardiovascular system, such as *EPAS1*, *SIRT1* and *EDNRA*, were genetically differentiated between highland and lowland populations of cashmere goats. However, no polymorphisms were found at the exons of *EPAS1* in our Tibetan cashmere goats, although a missense mutation in this gene was discovered in other Tibetan goat populations.

### Several outlier windows in the Boer goat overlap with selection signatures in other Boer populations

In this study, a total of 68 outlier windows, including 49 genes, were proposed to be under selection in Boer goats, based on both ZF_ST_ and ZH_p_ scores (Supplementary Dataset S3 and Table S2). Some of these regions overlapped with those identified in previous studies; for example, the outlier windows on chromosome 7 (44.6–44.7 and 58.45–58.60 Mb) and 13 (23.55–23.65 and 24.13–24.25 Mb) overlapped with selection signatures detected in Canadian Boer (39.1–62.4 Mb) and Australian Boer goats (20.9–40.1 Mb), respectively, in the study by Brito *et al*.^13^. As a meat breed, Boer goats are well-known for a fast growth rate, large body size and good carcass quality^2^. However, according to the functions of the 49 annotated genes in these regions, it seems difficult to establish associations of these regions with the growth- or body size-related traits in Boer goats. Similarly, the previous studies did not report any convincing selection signatures in other Boer populations^13,20^, possibly due to small sample sizes, low densities of SNPs, or the extent of genetic differentiation of the analyzed populations. Furthermore, this lack of signatures might suggest that selection for quantitative traits leaves little or no classic signatures of selection in domestic animals, which was supported by findings in eight cattle breeds^7^.

## Conclusions

Our results showed that a high degree of genetic diversity was present in our sample of domestic goat populations. In summary, our investigation identified many putative genomic regions under positive selection in the domestic goat genome. Some genes were likely associated with coat color, reproduction, and high-altitude adaptation traits in goats, reflecting phenotypic evolution under different selection goals and adaptation to the local environments.

## Electronic supplementary material

Supplementary information

Dataset S1

Dataset S2

Dataset S3
