# Supplementary material for: Whole-genome sequencing reveals selection signatures associated with important traits in six goat breeds
Source: Sci Rep. 2018 Jul 10;8:10405. doi: 10.1038/s41598-018-28719-w (PMC6039503; doi:10.1038/s41598-018-28719-w)
Supplement: Supplementary file 1 — Supplementary information [file 41598_2018_28719_MOESM1_ESM.pdf]

Whole-genome sequencing reveals selection signatures associated with important traits in six goat breeds

Jiazhong Guo[1], Haixi Tao[1], Pengfei Li[1], Li Li[1], Tao Zhong[1], Linjie Wang[1], Jinying Ma[2], Xiaoying Chen[2], Tianzeng Song[2*] & Hongping Zhang[1*]

[1]College of Animal Science and Technology, Sichuan Agricultural University, Chengdu, Postcode 611130, China

[2]Institute of Animal Science, Tibet Academy of Agricultural and Animal Husbandry Science, Lhasa, 850009, China

*Corresponding authors: T. S. (songtianzeng@chian.com.cn) or H.Z. (zhp@sicau.edu.cn)

This file includes: supplementary Figure S1 and supplementary Table S1-S7.

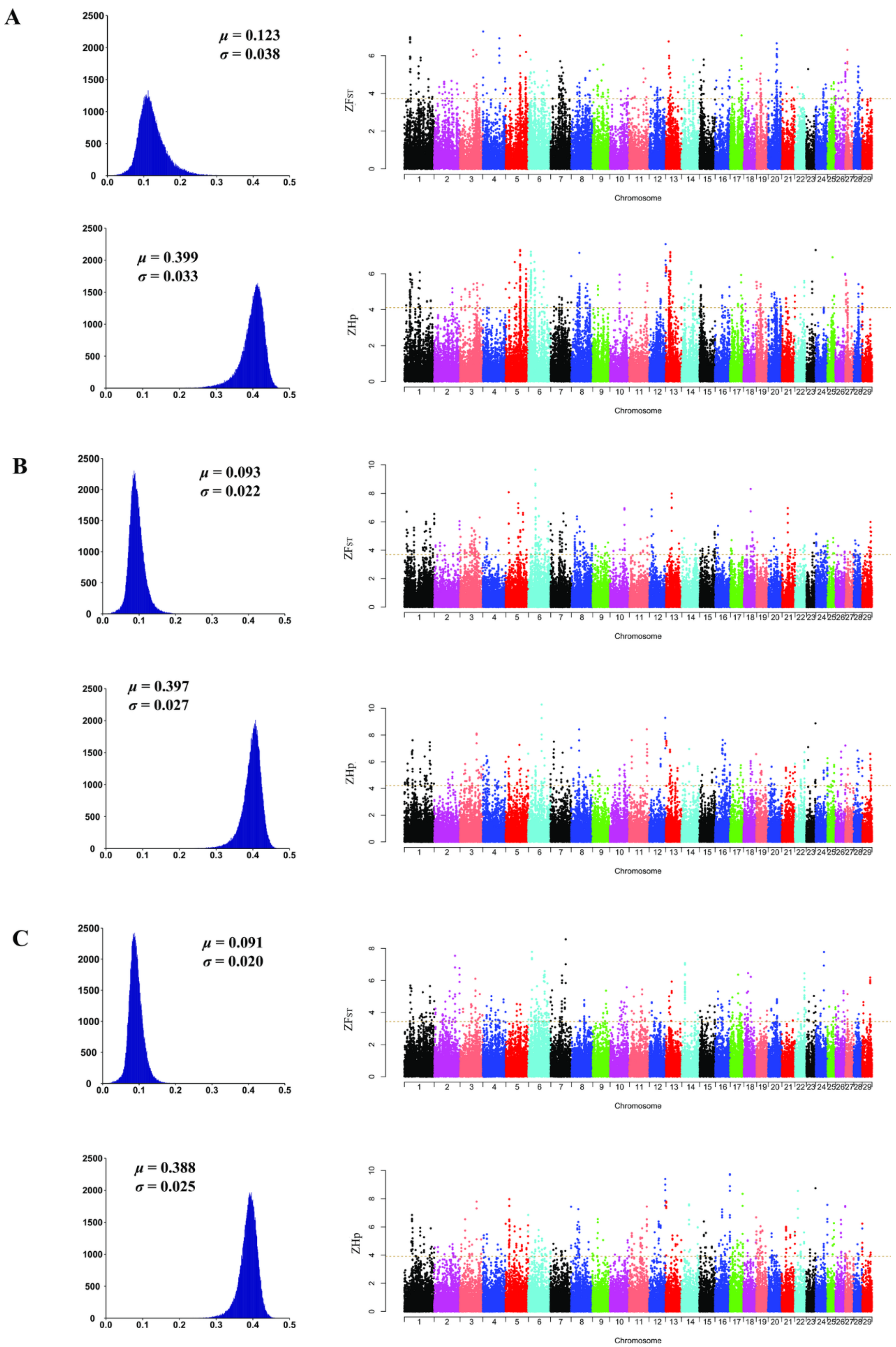

Supplementary Figure S1. Genome-wide distributions of selection signals in Boer (A), Jintang Black (B), and Tibetan goat (C). Manhattan plots of ZF_{ST} (> 0) and absolute values of ZHp (< 0) across all autosomes were plotted with different colors. ZF_{ST} and ZH_p values were calculated for

each sliding 100-kb window with a step of 25 kb across all autosomes. The horizontal dashed line indicates the 99.5 percentile of all ZF_{ST} or ZH_p values.

Supplementary Table S1. Summary of mapping results for clean reads in six goat breeds

Breed	Raw reads	Clean reads	Mapped reads (%)	Genome coverage	Sequence depth
Boer	476,423,532	476,163,564	461,493,427 (96.92)	99.88	23.68×
Meigu	501,029,630	500,519,978	483,840,905 (96.67)	99.87	24.30×
Jintang Black	529,288,018	445,976,744	433,745,404 (97.26)	99.88	22.30×
Nanjiang Yellow	446,245,326	528,760,500	510,974,229 (96.64)	99.87	26.35×
Tibetan	642,970,312	642,377,922	623,255,526 (97.02)	99.89	31.75×
Tibetan cashmere	587,395,048	582,825,190	556,617,458 (95.50)	99.89	27.35×

Supplementary Table S2. Genes within the overlapped selection regions identified via ZF_{ST} and ZH_p values in the Boer goats

Gene	Long Name	Chr	Position	ZF_{ST}^*	ZH_p^*
CADM2	Cell adhesion molecule 2	1	31,602,511-32,898,032	5.902	-5.947
LOC106502094		1	75,696,854-75,700,886	5.057	-5.208
CCDC50	Coiled-coil domain containing 50	1	75,702,789-75,778,927	4.085	-4.307
MTF2	Metal response element binding transcription factor 2	3	70,163,931-70,238,917	6.303	-5.426
TMED5	Transmembrane p24 trafficking protein 5	3	70,260,069-70,280,309	6.303	-5.426
PKP2	Plakophilin 2	5	75,562,425-75,654,587	4.104	-4.418
LOC102181103		5	75,666,788-75,667,765	4.062	-4.440
YARS2	Tyrosyl-tRNA synthetase 2	5	75,689,580-75,701,978	4.062	-4.440
DNM1L	Dynamin 1 like	5	75,702,304-75,764,731	4.062	-4.440
BICD1	BICD cargo adaptor 1	5	76,054,530-76,298,702	5.071	-4.287
TSPAN9	Tetraspanin 9	5	105,479,438-105,669,891	4.013	-5.002
LOC108636068		5	107,316,612-107,319,904	6.196	-5.747
LOC108636069		5	107,321,368-107,332,121	6.196	-5.747
CACNA1C	Calcium voltage-gated channel subunit alpha1 C	5	107,328,368-107,719,644	6.196	-5.747
ALPK1	Alpha kinase 1	6	13,456,823-13,570,751	5.117	-5.532
LOC102179747		6	13,549,965-13,551,080	5.117	-5.532
TIFA	TRAF interacting protein with forkhead associated domain	6	13,580,947-13,589,288	5.795	-5.414
AP1AR	Adaptor related protein complex 1 associated regulatory protein	6	13,595,155-13,624,310	5.795	-5.414

LOC108636214		6	35,356,486-35,359,054	4.506	-4.820
SNCA	Synuclein alpha	6	35,380,577-35,536,571	4.506	-4.820
LOC102174549		6	70,938,816-70,939,404	4.018	-4.708
LOC108636277		6	70,941,596-70,959,636	4.018	-4.708
LOC108636229		6	81,287,377-81,317,153	4.040	-6.133
HAND1	Heart and neural crest derivatives expressed 1	7	44,621,033-44,624,336	4.925	-4.703
SAP30L	SAP30-like	7	44,638,848-44,652,660	4.925	-4.703
GALNT10	Polypeptide N-acetylgalactosaminyltransferase 10	7	44,665,723-44,897,352	4.925	-4.703
LOC102181119		7	58,411,894-58,598,953	5.370	-4.654
LOC102180589		7	58,470,612-58,472,221	4.927	-4.278
LOC102186392		7	58,534,874-58,537,636	5.370	-4.654
LOC108636357		7	58,553,585-58,556,463	5.370	-4.654
LOC102187678		7	58,560,350-58,563,374	5.370	-4.654
LOC102187401		7	58,566,993-58,571,226	5.370	-4.654
LOC102183789		8	43,594,519-43,594,911	4.554	-5.132
KANK1	KN motif and ankyrin repeat domains 1	8	43,599,193-43,810,582	4.554	-5.132
LOC102183600		8	74,506,660-74,524,290	4.701	-4.362
APTX	Aprataxin	8	74,548,279-74,564,213	4.814	-4.397
DNAJA1	DnaJ heat shock protein family (Hsp40) member A1	8	74,588,289-74,599,055	4.814	-4.397
SMU1	SMU1, DNA replication regulator and spliceosomal factor	8	74,602,402-74,634,128	4.814	-4.397
ABI1	Abl interactor 1	13	17,149,896-17,235,134	3.750	-5.647
LOC102177812		13	17,190,396-17,191,806	3.750	-5.647
PDSS1	Decaprenyl diphosphate synthase subunit 1	13	17,234,961-17,274,832	3.750	-5.647
LOC102181052		13	17,274,925-17,303,119	3.750	-5.647
PARD3	Par-3 family cell polarity regulator	13	17,803,932-18,377,838	5.995	-4.630
PTF1A	Pancreas specific transcription factor, 1a	13	23,585,625-23,587,517	4.834	-4.896
NIPAL2	NIPA like domain containing 2	14	16,173,131-16,256,193	4.866	-4.337
LOC102179113		14	16,287,903-16,288,953	4.866	-4.337
ST18	ST18, C2H2C-Type zinc finger	14	60,814,276-60,905,059	5.016	-4.435
PCMTD1	Protein-L-isoaspartate (D-Aspartate) O-methyltransferase domain containing 1	14	61,001,518-61,049,881	5.766	-4.435
DNM3	Dynamin 3	16	37,786,098-38,414,194	4.262	-4.224

Note: The ZF_{ST} was the highest window ZF_{ST} value observed in the adjacent selection windows. The H_p value was the lowest window H_p value observed in the adjacent selection windows.

Supplementary Table S3. Genes within the overlapped selection regions identified via ZF_{ST} and ZH_p values in the Meigu goats

Gene	Long Name	Chr	Position	ZF_{ST}	ZH_p
LOC100861181		1	3,193,104-3,193,810	3.759	-4.791
KAP8	Keratin associated protein 8	1	3,215,311-3,215,883	3.759	-4.791
MECOM	MDS1 and EVI1 complex locus	1	97,221,943-97,849,739	6.972	-5.326
RYK	Receptor-like tyrosine kinase	1	134,654,014-134,765,283	4.895	-5.803
PRKAA2	Protein kinase, AMP-activated, alpha 2 catalytic subunit	3	31,225,506-31,309,357	6.057	-4.432
CDK14	Cyclin dependent kinase 14	4	111,953,410-112,626,647	4.916	-6.331
SPSB2	SPlA/ryanodine receptor domain and SOCS box containing 2	5	102,500,821-102,503,988	5.105	-5.240
TPI1	Triosephosphate isomerase 1	5	102,503,843-102,507,306	5.105	-5.240
USP5	Ubiquitin specific peptidase 5	5	102,508,238-102,521,693	5.105	-5.240
CDCA3	Cell division cycle associated 3	5	102,522,453-102,524,976	5.105	-5.240
GNB3	G protein subunit beta 3	5	102,526,716-102,534,103	5.022	-4.460
P3H3	Prolyl 3-hydroxylase 3	5	102,534,204-102,547,666	5.105	-5.240
GPR162	G protein-coupled receptor 162	5	102,548,646-102,554,523	5.105	-5.240
CD4	CD4 molecule	5	102,556,627-102,579,742	5.105	-5.240
LAG3	Lymphocyte activating 3	5	102,589,957-102,596,511	5.105	-5.240
PTMS	Parathymosin	5	102,597,981-102,602,830	5.105	-5.240
MLF2	Myeloid leukemia factor 2	5	102,615,233-102,620,477	5.022	-4.460
ARFGAP3	ADP ribosylation factor GTPase activating protein 3	5	112,591,791-112,642,935	4.191	-5.314
PACSIN2	Protein kinase C and casein kinase substrate in neurons 2	5	112,659,474-112,771,854	4.191	-5.314
LOC108636229		6	81,287,377-81,317,153	4.264	-4.917
SSBP2	Single stranded DNA binding protein 2	7	28,868,828-29,177,782	4.713	-5.606
TCF12	Transcription factor 12	10	49,776,513-50,167,172	4.245	-5.050
IL1R1	interleukin 1 receptor, type I	11	6,746,282-6,840,148	4.131	-6.049
STARD13	StAR related lipid transfer domain containing 13	12	58,686,583-59,067,985	5.391	-6.166
PTPRA	Protein tyrosine phosphatase, receptor type A	13	51,486,465-51,652,659	3.870	-5.103
VPS16	VPS16, CORVET/HOPS core subunit	13	51,659,722-51,686,297	3.870	-5.103

PCED1A	PC-esterase domain containing 1A	13	51,686,402-51,691,569	3.870	-5.103
LOC102186223		13	51,699,783-51,703,283	3.870	-5.103
RPN2	Ribophorin II	13	65,710,144-65,734,552	6.531	-5.396
LOC108637357		13	65,750,256-65,759,991	6.834	-5.396
GHRH	Growth hormone releasing hormone	13	65,769,415-65,778,809	6.834	-5.615
MANBAL	Mannosidase, beta A, lysosomal-like	13	65,803,119-65,827,734	6.834	-5.615
LOC108637372		13	65,831,607-65,840,196	6.834	-5.615
SRC	SRC proto-oncogene, non-receptor tyrosine kinase	13	65,852,275-65,906,216	4.840	-5.587
LOC108637679		15	49,279,392-49,570,289	4.721	-4.393
NECTIN1	Nectin cell adhesion molecule 1	15	52,079,830-52,152,192	6.900	-4.311
DISP3	Dispatched RND transporter family member 3	16	40,642,918-40,700,187	5.423	-4.777
LOC106502956		16	40,793,627-40,798,534	5.423	-6.518
UBIAD1	UbiA prenyltransferase domain containing 1	16	40,869,569-40,881,667	4.987	-6.518
MTOR	Mechanistic target of rapamycin kinase	16	40,889,820-41,015,393	4.747	-5.877
EXOSC10	Exosome component 10	16	41,023,287-41,045,517	5.257	-5.877
SRM	Spermidine synthase	16	41,062,640-41,066,649	5.257	-6.011
MASP2	Mannan binding lectin serine peptidase 2	16	41,080,217-41,096,199	5.985	-6.216
TARDBP	TAR DNA binding protein	16	41,097,063-41,103,700	5.985	-6.216
C16H1orf127		16	41,145,834-41,161,708	5.985	-6.216
ENO1	Enolase 1	16	43,110,101-43,124,014	6.546	-4.817
RERE	Arginine-glutamic acid dipeptide repeats	16	43,171,517-43,587,056	6.546	-4.864
LAMC1	Laminin, gamma 1	16	62,678,983-62,800,726	5.921	-5.721
CORO1C	Coronin 1C	17	6,813,088-6,891,975	7.906	-5.376
LOC106503038		17	6,882,430-6,883,376	7.906	-5.376
SSH1	Slingshot protein phosphatase 1	17	6,918,981-6,975,713	8.837	-6.112
DAO	D-amino acid oxidase	17	6,987,293-7,004,648	8.837	-6.112
MSI1	Musashi RNA binding protein 1	17	8,440,031-8,463,388	4.820	-5.437
PLA2G1B	Phospholipase A2 group IB	17	8,470,981-8,477,328	5.360	-5.437
SIRT4	Sirtuin 4	17	8,487,033-8,501,308	5.414	-5.614
LOC102178382		17	8,512,253-8,516,099	5.414	-5.614
PXN	Paxillin	17	8,515,969-8,558,916	6.610	-6.920
RPLP0	Ribosomal protein lateral stalk	17	8,561,764-8,565,824	6.610	-6.920

	subunit P0				
GCN1	GCN1, eIF2 alpha kinase activator homolog	17	8,567,265-8,621,749	6.610	-6.920
LOC108637861		17	8,621,857-8,631,848	5.679	-6.828
RAB35	RAB35, member RAS oncogene family	17	8,631,716-8,649,432	5.679	-6.828
BICDL1	BICD family like cargo adaptor 1	17	8,649,933-8,729,591	5.679	-6.828
SPIRE2	Spire type actin nucleation factor 2	18	16,045,124-16,078,337	4.804	-5.134
TCF25	Transcription factor 25	18	16,080,645-16,102,527	4.804	-5.134
LOC102181148		18	16,102,840-16,116,932	4.804	-5.134
DEF8	Differentially expressed in FDCP 8	18	16,121,580-16,138,058	4.804	-5.134
CENPBD1	CENPB DNA-binding domain containing 1	18	16,137,777-16,141,183	4.804	-5.134
LOC102181419		18	16,141,257-16,161,265	4.804	-5.134
DBNDD1	Dysbindin domain containing 1	18	16,162,963-16,170,801	4.804	-5.134
GAS8	Growth arrest specific 8	18	16,173,747-16,209,877	4.804	-5.134
CHMP6	Charged multivesicular body protein 6	19	51,032,923-51,040,342	5.192	-5.057
RPTOR	Regulatory associated protein of MTOR complex 1	19	51,050,316-51,373,104	6.492	-5.831
DNAH1	Dynein axonemal heavy chain 1	22	48,584,957-48,662,109	5.066	-4.982
MIR135A	MicroRNA 135a	22	48,686,064-48,686,153	5.066	-4.982
GLYCTK	Glycerate kinase	22	48,686,847-48,692,872	5.066	-4.982
WDR82	WD repeat domain 82	22	48,718,135-48,735,557	5.066	-4.982
MIRLET7G	MicroRNA let-7g	22	48,724,027-48,724,133	5.066	-4.982
PPM1M	Protein phosphatase 1M	22	48,737,453-48,742,007	5.066	-4.982
TWF2	Twinfilin actin binding protein 2	22	48,751,706-48,760,850	3.935	-4.344
TLR9	Toll like receptor 9	22	48,764,919-48,768,006	3.935	-4.344
LOC102180551		23	103,134-111,587	8.307	-5.872
DUSP22	Dual specificity phosphatase 22	23	111,611-159,290	8.307	-5.872
IRF4	Interferon regulatory factor 4	23	189,741-204,470	9.782	-6.965
EXOC2	Exocyst complex component 2	23	237,311-359,937	8.325	-4.847
KHDRBS2	KH RNA binding domain containing, signal transduction associated 2	23	47,872,421-48,672,920	6.159	-7.121

Supplementary Table S4. Genes within the overlapped selection regions identified via ZF_{ST} and ZH_p values in the Jintang Black goats

Gene	Long Name	Chr	Position	ZF_{ST}	ZH_p
LOC102180704		1	107,084,446-107,235,817	5.303	-6.398
LOC102175717		1	107,219,473-107,224,098	5.303	-4.949
LOC102178770		1	107,266,507-107,461,521	5.303	-6.398
MBNL1	Muscleblind like splicing regulator 1	1	114,974,326-115,192,808	4.105	-4.836
RYK	Receptor-like tyrosine kinase	1	134,654,014-134,765,283	5.499	-7.165
CHCHD3	Coiled-coil-helix-coiled-coil-helix domain containing 3	4	23,415,037-23,697,234	4.441	-6.125
CAMK4	Calcium/calmodulin-dependent protein kinase IV	7	80,527-352,060	5.848	-5.082
WDR36	WD repeat domain 36	7	469,008-513,272	5.392	-4.672
TGFBR1	Transforming growth factor beta receptor 1	8	63,759,178-63,829,333	4.530	-4.506
HNRNPC	Heterogeneous nuclear ribonucleoprotein C	10	76,993,485-77,038,760	4.467	-5.776
RPGRIP1	RPGR interacting protein 1	10	77,065,101-77,110,109	4.467	-5.776
LOC102172837		18	37,816,228-37,840,718	3.905	-5.891
NFAT5	Nuclear factor of activated T cells 5	18	37,925,539-38,044,038	4.260	-6.047
NLN	Neurolysin	20	13,756,180-13,851,801	3.943	-4.732
CACNA2D2	Clcium voltage-gated channel auxiliary subunit alpha 2 delta 2	22	49,928,448-50,067,976	4.250	-6.710
TMEM115	Transmembrane protein 115	22	50,070,038-50,074,820	4.250	-6.710
LOC102171017		22	50,075,651-50,078,335	4.347	-6.710
NPRL2	Nitrogen permease regulator-like 2	22	50,078,398-50,081,751	4.347	-6.710
ZMYND10	Zinc finger MYND-type containing 10	22	50,081,850-50,087,423	4.347	-6.710
RASSF1	Ras association domain family member 1	22	50,087,646-50,096,636	4.347	-6.710
TUSC2	Tumor suppressor candidate 2	22	50,097,265-50,100,349	4.347	-6.710
HYAL2	Hyaluronoglucosaminidase 2	22	50,102,362-50,107,589	4.347	-6.710
HYAL1	Hyaluronoglucosaminidase 1	22	50,110,568-50,113,767	4.347	-6.710
HYAL3	Hyaluronoglucosaminidase	22	50,114,244-50,119,914	4.347	-6.710

	3				
NAT6	N-acetyltransferase 6	22	50,114,480-50,117,170	4.347	-6.710
IFRD2	Interferon related developmental regulator 2	22	50,120,205-50,148,713	4.347	-6.710
LSMEM2	Leucine rich single-pass membrane protein 2	22	50,149,119-50,152,338	4.347	-6.710
SEMA3B	Semaphorin 3B	22	50,165,470-50,175,429	4.347	-5.546
GNAI2	G protein subunit alpha i2	22	50,180,963-50,201,171	3.986	-5.679
PRR35	Proline rich 35	25	429,477-433,159	4.646	-5.899
NHLRC4	NHL repeat containing 4	25	434,877-436,310	4.646	-5.899
PIGQ	Phosphatidylinositol glycan anchor biosynthesis class Q	25	436,361-449,696	4.646	-5.899
RAB40C	RAB40C, member RAS oncogene family	25	455,422-477,949	4.646	-6.262
WFIKKN1	WAP, follistatin/kazal, immunoglobulin, kunitz and netrin domain containing 1	25	478,071-481,893	4.646	-6.262
METTL26	Methyltransferase like 26	25	482,230-484,667	4.646	-6.262
MCRIP2	MAPK regulated corepressor interacting protein 2	25	484,781-496,540	4.646	-6.262
WDR90	WD repeat domain 90	25	496,629-511,801	4.646	-6.262
RHOT2	Ras homolog family member T2	25	511,991-517,346	4.646	-6.262
RHBDL1	Rhomboid like	25	518,296-521,352	4.646	-6.262
LOC106503559		25	521,543-522,939	4.646	-6.262
STUB1	STIP1 homology and U-box containing protein 1	25	523,059-525,430	4.646	-6.262
JMJD8	Jumonji domain containing 8	25	524,325-527,151	4.646	-6.262
WDR24	WD repeat domain 24	25	527,271-533,386	4.639	-6.262
FBXL16	F-box and leucine rich repeat protein 16	25	533,925-545,443	4.639	-6.262
C28H10orf35		28	19,717,910-19,721,038	3.690	-6.847
NEUROG3	Neurogenin 3	28	19,757,060-19,757,827	3.690	-6.847
NTM	Neurotrimin	29	33,839,843-34,799,485	4.212	-4.563
LOC102189031		29	42,293,614-42,318,571	3.745	-6.580
LOC108634205		29	42,319,845-42,338,237	3.745	-6.580
LOC102176672		29	42,345,355-42,381,309	3.745	-6.580
SCYL1	SCY1 like pseudokinase 1	29	44,121,687-44,132,769	5.317	-4.521
LTBP3	Latent transforming growth factor beta binding protein 3	29	44,131,694-44,152,416	5.317	-4.521
LOC102169263		29	44,154,996-44,159,072	5.317	-4.521
SSSCA1	Sjogren	29	44,168,506-44,172,674	5.317	-4.521

	syndrome/scleroderma autoantigen 1				
FAM89B	Family with sequence similarity 89, member B	29	44,170,943-44,172,674	5.317	-4.521
EHBP1L1	EH domain binding protein 1 like 1	29	44,174,580-44,190,648	5.317	-4.521

Supplementary Table S5. Genes within the overlapped selection regions identified via ZF_{ST} and ZH_p values in the Nanjiang Yellow goats

Gene	Long Name	Chr	Position	ZF_{ST}	ZH_p
LOC102168623		1	102,415,661-102,417,855	4.969	-4.249
LOC108636359		1	102,693,905-102,694,044	5.245	-4.584
LOC102179908		3	18,922,706-18,925,605	7.164	-4.252
DMAP1	DNA methyltransferase 1 associated protein 1	3	18,935,998-18,945,617	7.164	-4.252
ERI3	ERI1 exoribonuclease family member 3	3	18,946,003-19,078,626	7.164	-4.252
RNPC3	RNA binding region containing 3	3	80,656,949-80,688,529	5.665	-5.180
LOC102169641		3	80,732,412-80,757,937	4.868	-4.568
POLR3B	RNA polymerase III subunit B	5	68,406,602-68,521,308	5.721	-4.722
LDB2	LIM domain binding 2	6	111,976,958-112,438,346	4.883	-4.236
SCARA5	Scavenger receptor class A member 5	8	10,651,948-10,785,995	4.251	-4.208
GKAP1	G kinase anchoring protein 1	8	76,763,253-76,861,623	4.821	-4.597
RSPO4	R-spondin 4	13	59,419,545-59,456,637	5.293	-4.329
RALY	RALY heterogeneous nuclear ribonucleoprotein	13	62,969,108-63,053,806	4.100	-6.204
EIF2S2	Eukaryotic translation initiation factor 2 subunit beta	13	63,062,854-63,082,442	4.100	-6.204
SPIRE2	Spire type actin nucleation factor 2	18	16,045,124-16,078,337	5.207	-6.660
TCF25	Transcription factor 25	18	16,080,645-16,102,527	5.207	-6.660
LOC102181148		18	16,102,840-16,116,932	5.207	-6.660
DEF8	Differentially expressed in FDCP 8 homolog	18	16,121,580-16,138,058	5.207	-6.660
CENPBD1	CENPB DNA-binding domain containing 1	18	16,137,777-16,141,183	5.207	-6.660
LOC102181419		18	16,141,257-16,161,265	5.207	-6.660

DBNDD1	Dysbindin domain containing 1	18	16,162,963-16,170,801	5.207	-6.660
GAS8	Growth arrest specific 8	18	16,173,747-16,209,877	5.207	-6.660
RFFL	Ring finger and FYVE like domain containing E3 ubiquitin protein ligase	19	14,637,693-14,711,886	7.092	-4.899
LIG3	DNA ligase 3	19	14,713,967-14,735,131	6.929	-4.899
ZNF830	Zinc finger protein 830	19	14,753,732-14,756,613	7.092	-4.899
CCT6B	Chaperonin containing TCP1 subunit 6B	19	14,756,667-14,791,901	7.092	-4.899
FAM179B	Family with sequence similarity179 member B	21	54,230,794-54,306,821	4.745	-5.279
PRPF39	Pre-MRNA processing factor 39	21	54,310,873-54,345,507	4.745	-5.279
FKBP3	FK506 binding protein 3	21	54,345,749-54,356,109	4.745	-5.279
FANCM	Fanconi anemia complementation group M	21	54,357,589-54,418,209	4.745	-4.704
BCL11B	B-Cell CLL/lymphoma 11B	21	63,473,426-63,575,320	4.432	-5.146
CACNA2D2	Calcium voltage-gated channel auxiliary subunit alpha2delta 2	22	49,928,448-50,067,976	4.222	-4.524

Supplementary Table S6. Genes within the overlapped selection regions identified via ZF_{ST} and ZH_p values in the Tibetan goats

Gene	Long name	Chr	Position	ZF_{ST}	ZH_p
LOC102172205		1	135,138,133-135,177,945	5.649	-4.000
LOC102172488		1	135,201,755-135,247,066	5.649	-4.000
KAT2B	Lysine acetyltransferase 2B	1	157,085,938-157,185,530	4.709	-4.466
SGO1	Shugoshin 1	1	157,201,907-157,222,142	4.709	-4.466
LOC102178524		6	85,812,693-85,850,751	6.367	-4.474
LAGE3	L antigen family member 3	6	85,840,653-85,841,323	5.923	-4.447
LOC102169846		6	85,878,003-85,901,026	6.367	-4.474
EPGN	Epithelial mitogen	6	89,772,748-89,779,468	3.896	-4.522
EREG	Epiregulin	6	89,833,683-89,853,577	3.976	-4.522
LOC102180409		15	34,333,254-34,334,202	3.641	-5.586
LOC102173402		15	34,342,443-34,343,438	3.641	-5.586
LOC102173106		15	34,363,399-34,364,422	3.641	-5.586
LOC102172828		15	34,373,933-34,374,871	3.641	-5.586
LOC102180142		15	34,391,983-34,392,924	3.641	-5.586
LOC102172560		15	34,406,009-34,407,028	3.641	-5.586

LOC102172282		15	34,414,836-34,415,855	3.641	-5.586
LOC102179868		15	34,424,284-34,425,231	3.641	-5.586
LOC102173492		18	22,110,173-22,119,351	6.463	-5.003
C18H16orf87		18	22,119,396-22,154,779	6.463	-5.046
MYLK3	Myosin light chain kinase 3	18	22,188,697-22,250,039	6.463	-5.046
ZNF852	Zinc finger protein 852	22	16,189,711-16,202,009	3.599	-7.284
LOC102177570		22	16,204,212-16,284,793	3.963	-8.567
ZNF502	Zinc finger protein 502	22	16,287,427-16,297,792	3.963	-8.567
ZNF501	Zinc finger protein 501	22	16,309,551-16,310,546	3.963	-8.567
KIAA1143		22	16,318,453-16,327,891	3.963	-8.567
KIF15	Kinesin family member 15	22	16,327,926-16,386,693	3.577	-6.650
LDLRAD4	Low density lipoprotein receptor class A domain containing 4	24	43,647,271-43,808,160	7.781	-4.102
FAM210A	family with sequence similarity 210 member A	24	43,802,788-43,825,139	7.781	-4.102
RNMT	RNA guanine-7 methyltransferase	24	43,825,214-43,852,257	7.781	-4.102

Supplementary Table S7. Genes within the overlapped selection regions identified via ZF_{ST} and ZH_p values in the Tibetan cashmere goats

Gene	Long Name	Chr	Position	ZF_{ST}	ZH_p
EPHA6	EPH receptor A6	1	39,786,137-40,756,386	4.080	-5.355
LOC106503253		1	40,197,790-40,198,896	4.080	-5.355
SATB1	SATB homeobox 1	1	155,262,593-155,364,610	4.752	-4.067
RNPC3	RNA binding region containing 3	3	80,656,949-80,688,529	5.749	-5.165
LOC102177391		3	95,673,020-95,673,773	4.262	-6.481
LOC108635861		4	120,611,385-120,677,267	5.969	-4.653
KITLG	KIT ligand	5	18,044,632-18,151,924	6.720	-6.034
PAH	Phenylalanine hydroxylase	5	65,286,583-65,371,807	6.410	-4.730
C6H4orf22		6	95,489,192-96,199,327	7.320	-10.510
PDSS2	Decaprenyl diphosphate synthase subunit 2	9	29,902,485-30,182,481	4.302	-3.953
EPAS1	Endothelial PAS domain	11	28,318,780-28,411,296	6.329	-4.328

	protein 1				
PAX1	Paired box 1	13	40,260,236-40,270,605	5.452	-4.215
CNIH4	Cornichon family AMPA receptor auxiliary protein 4	16	26,021,184-26,039,178	5.581	-4.859
WDR26	WD repeat domain 26	16	26,044,756-26,085,641	5.581	-4.948
LOC102186708		16	26,104,629-26,115,683	4.316	-4.948
LOC100860961		16	26,121,947-26,122,713	4.316	-4.948
DNM3	Dynamin 3	16	37,786,098-38,414,194	5.369	-4.426
PRLR	Prolactin receptor	20	38,891,738-39,090,307	7.035	-3.950
